# Addressing knowledge gaps in evidence-based psychiatric treatment: insights from recent research

**DOI:** 10.1007/s00406-024-01914-9

**Published:** 2024-09-26

**Authors:** Theresa Halms, Alkomiet Hasan

**Affiliations:** 1 Department of Psychiatry, Psychotherapy and Psychosomatics, Medical Faculty, University of Augsburg, BKH Augsburg, Geschwister-Schönert-Str. 1, 86156 Augsburg, Germany; 2 DZPG (German Center for Mental Health), partner site München/Augsburg, Augsburg, Germany

Dear Editor,

We were very pleased to read the interesting and important article by Petzold et al. [1] recently published in *Eur Arch Psychiatry Clin Neurosci*, where the knowledge, attitudes, beliefs and behavior regarding physical activity among a sample of clinical psychologists in Germany have been evaluated. This study offers valuable insights, particularly highlighting the lack of formal training on recommending physical activity, despite the highly reported frequency of such recommendations among the psychologists surveyed. Most notably, the study reveals important findings about the level of knowledge concerning the effectiveness of various treatment options within this professional group. The results showed no significant differences in the perceived effectiveness of pharmacotherapy and electroconvulsive therapy (ECT) compared to physical activity among the participating psychologists. Significant differences in the perceived effectiveness were only found for bright light therapy and psychotherapy when compared to physical activity. Bright light therapy was considered less effective, while psychotherapy was rated as more effective than physical activity. In interpreting these results, it must be noted that the assessment of the effectiveness of different treatment options is likely to vary between different diagnoses, treatment settings and degrees of severity. Due to the general, setting- and disorder-unspecific evaluation of effectiveness, conclusions drawn from these results may be limited. Simultaneously, the reported response patterns do not follow the current evidence laid down in available guidelines.

Overall, these findings point to a concerning gap in familiarity with specific evidence-based treatment options and a potential bias towards one specific treatment (here: psychotherapy) in the study sample. As described in the awareness-to-adherence model by Pathman et al. [2], effective guideline-based treatment requires healthcare providers to be aware of guidelines, agree with their content, regularly apply recommendations, and adhere to them consistently. The results of Petzold et al.’s study indicate that the psychologists surveyed may lack some knowledge of treatment guidelines and the established efficacy and effectiveness of pharmacotherapy, the combined treatment of pharmacotherapy and psychotherapy or of ECT for a variety of psychiatric disorders.

We were recently able to identify a related pattern of bias in a cross-sectional study on the implementation status of the German S3 guideline schizophrenia [3]. We found significant discrepancies between guideline awareness and adherence, with notable differences across professional groups. Among the participating physicians, psychologists and psychotherapists, psychosocial therapists and nurses, approximately 40% were aware of the guideline and only 7% reported to adhere to the German schizophrenia guideline. Further, our results showed that physicians demonstrated significantly higher scores for agreement and awareness compared to all other professional groups. Similar to the results reported by Petzold et al. on the rating of the effectiveness of different treatment options, the surveyed psychotherapists and psychologists of our sample considered the recommendation concerning psychotherapy in the schizophrenia guideline to be more appropriate and feasible for the treatment of patients with schizophrenia than all other professional groups. Further evidence of professional group-specific differences in knowledge of evidence-based treatment emerged from our recently published cluster-randomized trial [4]. Our findings showed that while 80% of psychiatrists passed the knowledge test, only 20% of the surveyed psychotherapists successfully completed the test. The knowledge-attitude bias demonstrated across studies and the overall low values for guideline adherence represent a significant barrier to the provision of optimal care for those affected.

One approach to increasing healthcare professionals’ knowledge and long-term adherence to evidence-based and guideline-compliant treatment of patients with psychiatric disorders is structured guideline implementation. Our cluster-randomized controlled trial demonstrated that a structured implementation of the German schizophrenia guideline leads to a significant increase in guideline knowledge among all physicians and psychologists. The structured implementation of guidelines should include reminders on the application of the guideline, the opportunity to discuss open questions with experts and the joint discussion of clinical cases to align with guideline recommendations. Continuous implementation efforts and frequent updates to guidelines based on the latest evidence are essential to improving knowledge across all professional groups, thereby ensuring that patients receive the most effective care. Finally, the training of guideline competence should be integrated in teaching concepts of medical and psychology students and in concepts for specialization after graduation. In doing so, all evidence-based treatment options, including exercise therapy for e.g. depression or schizophrenia, will be offered to patients.

## References

[1] Petzold MB, Betzler F, Plag J, Ströhle A, Bendau A (2024) Advising activity-knowledge, attitudes, beliefs, and behaviors regarding the recommendation of physical activity in clinical psychologists. European archives of psychiatry and clinical neuroscience. 10.1007/s00406-024-01819-7 10.1007/s00406-024-01819-7 PMC11362258 38714563

[2] Pathman DE, Konrad TR, Freed GL, Freeman VA, Koch GG (1996) The awareness-to-adherence model of the steps to clinical guideline compliance. The case of pediatric vaccine recommendations. Med Care 34(9):873–889. 10.1097/00005650-199609000-00002 8792778 10.1097/00005650-199609000-00002

[3] Khorikian-Ghazari N, Lorenz C, Güler D, Halms T, Röh A, Flick M, Burschinski A, Pielenz C, Salveridou-Hof E, Schneider-Axmann T, Schneider M, Wagner E, Falkai P, Gaebel W, Leucht S, Hasan A, Gaigl G (2023) Guideline for schizophrenia: implementation status and attitude toward an upcoming living guideline. Eur Arch Psychiatry Clin NeuroSci 273(7):1587–1598. 10.1007/s00406-023-01568-z 36808533 10.1007/s00406-023-01568-z PMC10465681

[4] Halms T, Gaigl G, Lorenz C, Güler D, Khorikian-Ghazari N, Röh A, Burschinski A, Gaebel W, Flick M, Pielenz C, Salveridou-Hof E, Schneider-Axmann T, Schneider M, Wagner E, Falkai P, Lucae S, Rentrop M, Zwanzger P, Seemüller F, Landgrebe M, Ortner M, Schneeweiß B, Brieger P, Ajayi K, Schwarz M, Heres S, Marstrander N, Becker T, Jäger M, Putzhammer A, Frasch K, Steber R, Leucht S, Hasan A (2024) The impact of a digital guideline version on schizophrenia guideline knowledge: results from a multicenter cluster-randomized controlled trial. BMC Med 22(1):311. 10.1186/s12916-024-03533-6 39075458 10.1186/s12916-024-03533-6 PMC11287881

